# Reliability, Validity and Factor Structure of the 12-Item General Health Questionnaire among General Population

**DOI:** 10.3889/oamjms.2015.075

**Published:** 2015-06-25

**Authors:** Miodraga Stefanovska Petkovska, Marjan I. Bojadziev, Vesna Velikj Stefanovska

**Affiliations:** 1 *University American College, Skopje, Republic of Macedonia*; 2 *Institute of Epidemiology and Biostatistics with Medical Informatics, Medical Faculty, Ss Cyril and Methodius University of Skopje, Skopje, Republic of Macedonia*

**Keywords:** General Health Questionnaire, factor structure, reliability, psychological health

## Abstract

**AIM:**

The aim of the study is to analyze the internal consistency; validity and factor structure of the twelve item General Health Questionnaire for the Macedonian general population.

**MATERIAL AND METHODS:**

Data came from nationally representative sample of 1603 randomly selected Macedonians all aged 18 years or older.

**RESULTS:**

The mean GHQ score in the general sample was found to be 7.9 (SD = 4.3). The results revealed a higher GHQ score among women (M = 8.91, SD = 4.5) compared to men (M = 6.89; SD = 4.2). The participants from the rural areas obtained a lower GHQ score (M = 7.55, SD = 3.8) compared to participants coming from the urban areas (M = 9.37, SD = 4.1). The principal component analysis with oblique rotation (direct oblimin) with maximum likelihood procedure solution was performed and the results yielded a three factor solution which jointly accounted for 57.17% of the total variance: Factor I named social management (items 1, 3, 4, 6, 7 and 8); Factor II stress (items 2, 5 and 9) and Factor III named self-confidence (items 10, 11 and 12). Its factor structure is in line with representative research from other population groups.

**CONCLUSION:**

The GHQ-12 can be used effectively for assessment of the overall psychological well-being and detection of non-psychotic psychiatric problems among the Macedonian population.

## Introduction

Mental health is an important indicator of the general health status in one society and its burden is considered as one of the most common causes of disability in the world [1]. According to the latest World Health Organization (WHO) Mental Health Action Plan 2013-2020, between 76% and 85% of people with severe mental disorders receive no treatment for their disorder in low-income and middle-income countries such as Macedonia; in parallel the corresponding range for high-income countries is between 35% and 50% [2]. An uprising problem in low-income and middle-income countries is the existing gap between the need, provision and quality of treatment for patients as well as the insufficient number of health personnel dealing with mental health problems or trained in the use of psychosocial interventions. In addition the numbers indicate that the awareness of public authorities is unfavourable as well - only 36% of people living in low income countries are covered by mental health legislation. In Republic of Macedonia, neuropsychiatric disorders are estimated to contribute to 19.5% of the global burden of disease. The suicide rate for males is 9.5 per 100.000 populations and for females are 4 per 100.000 populations. Taking into account its economic and social consequences, which do not just affect the individual but the society as a whole, WHO highlights the need for psychiatric prevention.

There are a variety of screening instruments that are able to diagnose and determine mental health disorders. However most of them have received validation only in developed economies and do not reflect the reality of the low and middle income economies, thus undermining the potential to do good quality research. This research reports on the translation and validation of 12 item General Health Questionnaire (GHQ 12) among the general population in Macedonia. The General Health Questionnaire was developed as a self-administered screening instrument aimed at detecting individuals with diagnosable mental health disorder [3] or to ‘differentiate psychiatric patients as a class from non-cases as a class [4]. It allows for distinguishing patients suffering from psychiatric problems from those in good mental health [5]. It can be administered among the general population and non-psychiatric settings [6]. The original version of this questionnaire was consisted of 60 items; however other versions with 30, 28 and 12 questions were also developed.

The GHQ 12 is the most widely used screening instrument for common mental disorders. It is recommended as a case detector since it is characterized with brevity, effectiveness and robustness and works as well as its longer versions [7]. The longer versions should be used only if there is an interest for scaled scored in addition to the total score. The GHQ-12 has been translated to many languages and its psychometric properties have been studied among many different populations groups including youth, elderly or urinary patients [8-18]. There are two different scoring styles of the questionnaire: Likert type (0-1-2-3) and bimodal (0-0-1-1).

Regarding the factor structure of the GHQ-12, the literature contains evidence supporting unidimensional, two-factor and three-factor conceptualizations of the GHQ-12 [16]. Analyses of the factor structure of the GHQ-12 by Corti [19] indicated a unidimensional model. Similar findings were confirmed by other researchers as well [20-22]. However other studies suggest that the GHQ-12 contains two and three clinically meaningful factor solutions [23-25]. In example, Politi et al. [24] extracted two factors named general dysphoria and social dysfunction. Based on his research among 6151 Australians aged 16-25, Graetz [23] reported a three factor structure. Factor I was called Anxiety (consisted of items 2, 5, 6, 9); factor II is Social dysfunction (consisted of items 1, 3, 4, 7, 8, 12) and factor III is Loss of confidence (consisted of items 10, 11). Lopez and Dresch [11] report a three factor structure in which Factor I is called Successful Coping (items 1, 3, 4, 7, 8, 12); factor II is called Self-esteem (items 6, 9, 10, 11) and Factor III is called Stress (items 3, 5, 9). The Tamil version of the GHQ-12 revealed three factors as well, which in accordance to the item content in each of the factors were named Depression-anxiety, Social performance and Self-esteem [26]. Another three-factor model emerged from a study that examined the validation of the GHQ 12 among the elderly population [27], more specifically 2123 participants aged over 60 years and 7490 younger adults in the United Kingdom. The study confirmed that the three factor structure proposed by Graetz had the best fit for younger and older people. In line with this the three factors obtained were Anxiety and Depression, Social Dysfunction, and Loss of Confidence. Since the GHQ-12 is effective, affordable, and brief and with a well documented research application as a psychiatric screening toll, it was decided to translate the instrument in Macedonian and administer it to a general population sample.

The aim of the study is to analyze the internal consistency; validity and factor structure of the twelve item General Health Questionnaire for the Macedonian general population.

## Material and Methods

### Instrument

The twelve-item GHQ (GHQ-12) consists of 12 items that assess the extent of a mental problem over the past few weeks. In order to obtain an overall GHQ score, responses were scored using a Likert type scale (0-3) instead of the binominal (0-0-1-1). A GHQ score is calculated for each subject by summing the scores for each of the twelve items. This total score can range from 0 (substantial decrease in all symptoms) to 36 (substantial increase in all symptoms). Higher scores indicate more psychiatric morbidity.

The validity was confirmed using the Subjective Vitality Scale (VS). This is a seven-item instrument developed by Ryan and Fredrick to measure vitality [28]. It has two versions: an Individual Difference Level Version, which asks individuals to respond to each item by indicating the degree to which it is generally true in their lives; and the State Level Version, which asks individuals to respond to each item in terms of how they are feeling at that moment. For the purpose of the research the individual difference level version of the vitality scale was used [29]. This instrument has previously been used to measure the relationship between psychological distress and vitality [31, 32].

The instrument was translated from English to Macedonian using the procedure of forward- backward translation procedure. With intention to develop a Macedonian version of the instrument that is conceptually equivalent to the original version, the back-translated English version was compared and discussed. The draft version was pilot tested and any identified discrepancies were discussed and solutions were reached through a consensus.

### Sample

The survey was administered on a random selected representative sample of the Macedonian general population aged above 18 years. The sample was calculated using data from the National Statistical Office (2002 state census) for population aged above 18 years, in regards to age, gender, education, municipality of residence and ethnicity. The research consisted of: 1) phase one - determining the number of representatives above 18 years of age from each of the 72 municipalities at national level and 2) phase two - administering the questionnaires in a one hour session in each of the municipalities in the country. The participants were invited to participate in one hour session. Members of the research team explained the purpose of the study and administered printed versions of the questionnaires to a randomly selected sample of 2504 individuals, out of which 1603 individuals agreed to participate. The participation was voluntary and the information on the purpose of the survey was explained on the cover page of each questionnaire. The survey was anonymous and no marks were printed on the questionnaires that could identify the respondent. All participants in the survey provided informed consent for their participation. After the questionnaire was completed, it was returned in an especially dedicated box by the participant. The methodological approach for administering the questionnaire has been based on previous research done in the field [11].

### Statistical analysis

The internal reliability of the GHQ-12 was assessed using the Cronbach’s alpha coefficient. Items that scored equal or greater than 0.70 were included in the further analysis. The sampling adequacy was detected trough Keiser-Meyer-Olkin (KMO) and Bartlet’s tests of spherecy. The factor structure of the GHQ-12 was determined trough principal component analysis with oblique rotation (direct oblimin) with maximum likelihood procedure solution. This was done in accordance to the recommendations by Graetz that oblique rotation(direct oblimin) with maximum likelihood procedure are more convenient and can deliver a solution that is simpler and easier to interpret, compared to the undesirable properties of the orthogonal (varimax) rotation. Convergent validity was evaluated in order to investigate the relationship between the twelve item version of the GHQ-12 and the VS. In addition, the questionnaire also collected the demographic information of the respondents (age, gender, education level, residence and region).

## Results

Completed questionnaires were collected from 1603 individuals from Republic of Macedonia with a sound response rate of 64.05%. Table 1 presents the demographic profile of the survey population.

**Table 1:** Demographic profile of survey population

**Gender**	%	(N)
Male	48.5	777
Female	51.5	826
**Age**		
18-26 years	22.2	356
27-37 years	30.1	483
38-54 years	28.8	462
55-64 years	15.3	245
Over 65 years	3.6	58
**Ethnicity**	**%**	**(N)**
Macedonian	69.3	1111
Albanian	24.9	399
Turkish	1.3	21
Roma	1.1	18
Serbian	1.3	21
Vlach	0.7	11
Other	1.4	22
**Residence**	**%**	**(N)**
Rural	63.3	1015
Urban	36.7	588
**Region**	**%**	**(N)**
Skopje	30.1	483
Southeast	9.1	146
East	8.8	141
Northeast	9	144
Vardar	7.3	117
Southwest	11	176
Polog	15.6	250
Pelagonija	9.1	146
**Education**	**%**	***(N)***
Primary school	19.6	314
High school	54.4	872
University	26	417

The GHQ mean score in the sample was found to be 7.9 (± 4.3). The mean score among women (8.91 ± 4.5) was higher compared to men (6.89 ± 4.2). The difference was found to be statistically significant (t = 5.1; p < 0.000). The participants from the rural areas obtained a lower GHQ score (7.55 ± 3.8) compared to participants from the urban areas (9.37 ± 4.1) with no significant differences between the two groups (p > 0.05).

The analysis related to internal consistency, showed the satisfactory results with Cronbach’s alpha above 0.70 (Table 2). All corrected item-total coefficients met the criterion value >0.20, with item “Felt you could not overcome your difficulties?” being the one with the lowest (0.34) and item “Felt constantly under strain?” with the highest coefficient (0.58) (Table 2).

**Table 2:** Results of Item-scale analysis

Item-scale	Adjusted Item scale correlation*	Cronbach alpha if eliminated
1. Able to concentrate on what you are doing?	0.35	0.82
2. Lost much sleep over worry?	0.44	0.84
3. Felt that you are playing useful part in things?	0.41	0.84
4. Felt capable of making decisions about things?	0.45	0.84
5. Felt constantly under strain?	0.58	0.82
6. Felt you could not overcome your difficulties?	0.34	0.85
7. Been able to enjoy your normal day to day activities?	0.41	0.84
8. Been able to face up to your problem?	0.52	0.81
9. Been feeling unhappy or depressed?	0.53	0.81
10. Been losing confidence in yourself?	0.48	0.84
11. Been thinking of yourself as a worthless person?	0.38	0.81
12. Been feeling reasonably happy, all things considered?	0.37	0.81
**Internal consistency of GHQ-12**	**Alpha**	**Standardized Alpha**
Whole sample	0.86	0.88
Men (*N=777*)	0.85	0.86
Women (*N=826*)	0.8	0.82
Rural areas (*N=1015)*	0.75	0.78
Urban areas *(N=588)*	0.71	0.79

**Figure 1:**
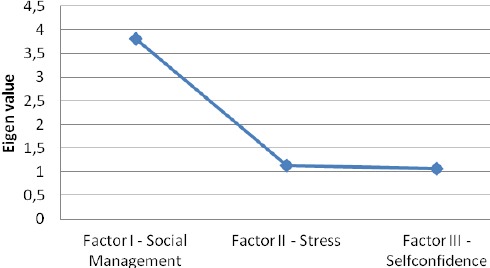
*Eigen values of each factor*.

The principal component analysis with oblique rotation (direct oblimin) with maximum likelihood procedure solution was performed and a three-factor structure was loaded. The sampling adequacy was detected trough Keiser-Meyer-Olkin (KMO = 0.83) and Bartlet’s tests of spherecy (P < 0.000). The factor structure of the GHQ-12, eigen values and the percentage of explained variance of each of the three factors are shown in Table 3 and Figure 1.

**Table 3:** Factor structure of GHQ-12 using oblique rotation (direct oblimin) with maximum likelihood estimates

	GHQ-12 Questions	Factor I Social management	Factor II Stress	Factor III Self confidence
1	Been able to concentrate on what you are doing?	0.49		
2	Lost much sleep over worry?		0.63	
3	Felt that you are playing useful part in things?	0.58		
4	Felt capable of making decisions about things?	0.67		
5	Felt constantly under strain?		0.78	
6	Felt you could not overcome your difficulties?	0.5		
7	Been able to enjoy your normal day to day activities?	0.71		
8	Been able to face up to your problem?	0.69		
9	Been feeling unhappy or depressed?		0.71	
10	Been losing confidence in yourself?			0.63
11	Been thinking of yourself as a worthless person?			0.65
12	Been feeling reasonably happy, all things considered?			0.5
	Eigen values	3.81	1.14	1.06
	Variance explained (%)	33.13	14.01	10.03
	Inter-factor correlations	Factor I	Factor II	Factor III
	Factor I	1		
	Factor II	-0.45	1	
	Factor III	0.19	-0.28	1

Three main factors were obtained: Factor I “Social management”, Factor II “Stress” and Factor III “Self-confidence”. The results showed that Factor I is a major factor that accounts for 33.13% of the variance, while Factor II and Factors III are minor factors that account for 14.01% and 10.03% of the variance respectively. All three factors jointly accounted for 57.17% of the variance. Finally the results suggested a moderate inter-correlation between the three factors. Namely, a higher correlation may be observed between Factor II-Stress and Factor I – Social management, while the lowest is between Factor I-Social Management and Factor III – Self-confidence.

The validity of the GHQ-12 was confirmed using the convergent validity method (Table 4). As expected, the results indicated a significant negative correlation between GHQ-12 and VS total scores (r = -0.77; p<0.01). This indicated that respondents which are more distressed have lower levels subjective vitality.

**Table 4:** Pearson correlation between GHQ-12 and VS

Variable		1	2	3	4	5
1	VS- Total score	1				
2	GHQ-12 Stress	-0.21	1			
3	GHQ-12 Soc.Man.	-0.33	0.3	1		
4	GHQ-12 SelfConf.	-0.18	0.01	-0.22	1	
5	GHQ-12-Total score	-0.77**	0.75**	0.64**	0.41**	1

* p<0.05 (2-tailed)

** p<0.01 (2-tailed)

## Discussion

The research paper reports the results of the validation study of the Macedonian translation of the GHQ-12. The reliability of the GHQ-12 in the general Macedonian population was 0.86; standardized alpha was 0.88, which supports the GHQ-12 as internally reliable. The comparison with indices found in other population’s suggested that the results are within the acceptable values. In example a study done among Japanese adults reported 0.83 for men and 0.85 for women, while a study done among Spanish population reported 0.76 for men and 0.70 for women [33, 11]. Another study reported Cronbach’s alpha of 0.78 for primary care attendants in Turkey and the GHQ-12 that was validated among the Arabic student population showed an alpha of 0.86 [34]. Results indicated small gender difference, however with slightly higher scores for women (women Cronbach’s alpha value was 0.8. standardized alpha 0.82; for men 0.85, standardized alpha 0.86). In addition the results indicate that every item is correlated with the scale total omitting that score.

This research suggests a three dimensional factorial structure of GHQ-12 among the Macedonian general population. In accordance to the item content in each of the three factors, Factor I is named *Social management* and included items 1, 3, 4, 6, 7 and 8; Factor II is named *Stress* and includes items: 2, 5 and 9; finally Factor III is named *Self-confidence* and included items 10, 11 and 12. In comparison with the factor structure obtained by Lopez and Dresch [11] the main difference is in the order of the factors. Factor I in both studies is equivalent and Factor II in their study equals Factor III in our study. In comparison to Graetz [23] the differences are found in the factor structure – Factor I in this study is equal to Factor II in their study. Factor III is equivalent in both studies. In our study Social dysfunction, Stress and Self-confidence accounted for 33.13%, 14.01% and 10.03% respectively of the variance explained; while in Graetz research anxiety, social dysfunction and loss of confidence accounted for 37%, 12% and 8% respectively of the explained variance. Werneke, Goldberg, Yalcin and Ustun [35] investigated combined data in 15 countries and reported that items 2 and 5 always loaded together and in many cases with the items 9 and 10. In this study items 2, 5 and 9 loaded together, but item 10 loaded on a separate factor. According to Minowa [33] the remaining factors designated as social dysfunction, social management, confidence etc., were subjects to variation possibly due to being more under the influence of the socio-cultural norms and values that govern the population under study.

In summary, this study provided an opportunity to validate the GHQ-12 in a randomly selected sample of the Macedonian general population. The results from the study suggest that the GHQ-12 is an internally reliable instrument and valid for use among this population. It contains three factors social management (items 1, 3, 4, 6, 7 and 8) stress (items 2, 5 and 9) and self-confidence (items 10, 11 and 12) and its factor structure is in line with representative research from other population groups. Consequently, the GHQ-12 can be used effectively for assessment of the overall psychological well-being and detection of non-psychotic psychiatric problems among the Macedonian population.
